# Gloucester Infirmary

**Published:** 1829-10-01

**Authors:** 


### CLINICAL REVIEW.

### IX.

### GLOUCESTER INFIRMARY.

#### DR. BARON ON IODINE.

In a late number of the Midland Reporter, Dr. Baron has stated some notes on the use of iodine, which deserve the practitioner's attention. This most potent of all "absorbents," or stimulants of the absorbents, will probably come into much more extended use than at present—and for many other diseases than those to which it seems now applicable. We are informed by a respectable practitioner, that he finds the tincture of iodine a most powerful expectorant, in those cases where expectoration is desirable, as in pneumonia, asthma, &c. This hint may be worth attending to.

*Case* 1. The first case which Dr. Baron states, was that of a girl, 15 years of age, who came to the infirmary on the 5th April, 1828. She was extremely emaciated — abdomen distended and fluctuating, the fluctuation having succeeded peritonitis. Powerful diuretics were given, and iodine ointment rubbed over the abdomen, but no amendment ensued. The effusion increased, and paracentesis was performed. The effusion returned, and the iodine ointment was resumed. It was continued steadily for two months, and under its administration the whole of the second effusion was absorbed. The only other remedies used were leeches to the anus, and a mixture of rhubarb, taraxacum, and magnesia. The general health improved, and quinine was given after the iodine was left off.

*Case* 2. This was a man, about 35 years of age, and an intemperate liver. He entered the infirmary in a state of the utmost exhaustion and emaciation. His respiration was impeded—abdomen tumid—extremities livid—tongue florid red — inappetency — frequent hæmorrhages from mouth and throat—had been tapped once before he came under Dr. B.'s care.

"My first object was to endeavour to improve his general health; to restrain the discharge of blood, and to act on the kidneys. I soon found, however, that little benefit was to be looked for in these respects, while the abdomen continued as it was. He was therefore tapped, and a very large quantity of serum was withdrawn. After this operation, an enlarged and hardened liver was very perceptible both to the touch and sight. I then recommenced what had been previously adopted without benefit, I mean the use of the iodine ointment, and a mixture with sulphate of potass and extract of taraxacum, together with the nitrate of potass and infusion of rhubarb. To these remedies were afterwards added some blue-pill, with squill-pill and extract of conium. The treatment of the complaint was chiefly conducted by these means, with occasional variations, all of which I need not specify. It is necessary however, to remark, that the abdomen filled very soon after the second tapping. Under the remedies above-mentioned, absorption began to take place, together with a manifest improvement in the nutritive process. His appetite amended, and he began to acquire flesh. While under my care, he had two attacks of peritoneal inflammation, during which the swelling of the abdomen increased. In both instances it was necessary to have recourse to the lancet. This man, from first to last, was about eight months in the Infirmary. Before he went out, the ascites was completely removed; the enlargement of the liver was much reduced, and, with the exception of this circumstance, and some slight œdema of the lower extremities, there was no trace of his former disease. The patient thought himself quite well, and left the house contrary to my wishes. I was anxious that he should continue in the use of remedies which had been so efficient, in the full expectation that the remaining affection of the liver, and the swelling of the feet, would have been altogether removed."

*Case* 3. The third case is different from both the foregoing. It was a boy, 15 years of age, who exhibited a well marked example of tuberculous disease of the serous membranes. The abdomen was tumid and hard, affording a distinct sense of fluctuation in some places. A hard and distinct tumour, the size of a half crown, was felt below the margin of the ribs on the right side. The skin was rough and scaly. The patient was much emaciated—suffered much distress after eating—and vomited almost every thing he swallowed. In addition, he shewed evident marks of thoracic disease—the breathing being oppressed, with pain on full inspiration. He had been ill six months, and mercury had been employed to remove the tension and tumefaction of the abdomen; but the disease had regularly gained ground. After endeavouring to allay the disorder of the stomach, and to regulate the bowels by small doses of aloes and opium, Dr. B. ordered the iodine ointment to be assiduously rubbed on the abdomen, night and morning, doing every thing in his power to promote the nutrition of the body and the absorption of the diseased parts. The liquor potassæ, in a bitter infusion, was frequently exhibited. In the course of the disease, various remedies were used, and leeches were sometimes necessary; but the iodine was evidently the efficient medicine. It was persevered in for more than 18 months; and, although the disease, at times, appeared to set all means at defiance, the ultimate success was complete. Every trace of abdominal disease vanished in the end, and the patient was restored to perfect health.

*Case* 4. A child, 18 months, old was brought to our author, with enlarged and, indurated abdomen — chest very narrow—all the other parts of the body much emaciated—flesh soft and flabby —nutrition almost stopped—as almost all solid food passed undigested. Our author recommended the abdomen to be rubbed twice a day with iodine ointment, and soda, tartrite of iron, and colomba to be taken internally. In a short time the abdomen became less hard and tumid—the diarrhœa ceased —the evacuations became more natural —while the countenance and temper of the child improved. In a short time more the thorax expanded—the flesh became less flabby, and the child was able to walk.

Dr. B. thinks he has good reason for believing that tuberculous diseases of the lungs, in their incipient stages, have been removed by treatment similar in principle to the above. In such cases, he has used the iodine ointment combined with opium, and directed it to be rubbed upon the thorax. Other means were, of course, employed; but he does not think they would have been effectual without the iodine.

"Allow me, now, to select two other cases, in which the most gratifying success has arisen from the use of the iodine. One was that of a female nearly forty years old. A tumour about the size of a child's head occupied the lower portion of the abdomen, and pressed on the pelvic viscera. Neither the fæces nor urine could be discharged without the greatest difficulty. It was so great as to require the frequent use of the rectum-bougie, as well as of the catheter. Besides these means of temporary relief, leeches to the anus, anodynes, fomentations, &c. were occasionally had recourse to. The iodine ointment was, in addition, assiduously employed. Towards the conclusion of the treatment, I directed the patient to take a solution of the chloride of lime; prior to its use, a decrease in the size of the tumour was rendered manifest, on examination, as well as by less frequent need for the bougie. The patient was under treatment in the Infirmary for many months. The disorganization, I am happy to say, has been so completely removed, that no trace of it can be felt externally, and all the inconvenience and pain which she suffered from it, have nearly disappeared.

"I have lately treated another case, of the same description as the preceding. The disease had, however, made greater progress. The tumours not only pressed upon the pelvic viscera, and impeded their functions; they likewise expanded into the abdomen, and produced all the distress consequent on this sort of disorganization. It was manifestly composed of different textures; some portions being hard, and others soft; and the surface of the abdomen affording that unevenness which denotes the origin and the character of the disease. In this case, the distress arising from the pressure of the tumour upon the bladder, was so great, that it was necessary to employ the catheter every day. Under these unpromising circumstances, the use of the medicine of which I have been speaking, was begun. It was employed both internally and externally for a considerable time. The result has been a great reduction of the tumour, complete freedom of the urinary organs, and such an improvement in the health of the patient, as to permit her to perform the arduous duties of an active situation with ease."

Dr. B. introduces some pathological observations on the evolution and progress of morbid structures; but with his opinions on these subjects our readers are well acquainted. The facts of the paper we have here recorded, as the most valuable portions of it.

### XXXVI.

### GLOUCESTER INFIRMARY.

I TUMOUR GROWING FROM THE DURA MATER AT THE BASIS CRANII.

Mr. Wood, who signs himself, late pupil to Mr. Fletcher, has reported two or three cases from the above institution, in the last Number of our Midland contemporary. The first case is not devoid of interest.

*Case.* "William Bevan, æt. 21, four months previous to his admission into the Infirmary, caught a violent cold, by working in a damp room under ground, which continued about a month, and then left him; but a few days afterwards, he experienced a dull aching pain in his right ear and temple, which gradually extended over his cheek, always continuing, but sometimes much worse than at others. He was then bled, and had leeches applied to his temple, by which the pain was a little alleviated. The pain then extended along his lower jaw, his right eye-lids were at the same time drawn a little outwards, which was accompanied by a sensation of stiffness.

"In proportion as the pain descended and spread over his face, the feeling of the affected parts diminished. He lost his hearing on that side entirely; the corner of his mouth was drawn towards his cheek, and his speech became very thick and indistinct. The inside of his mouth was a little swollen near the angle of the jaw; his taste on the right side was destroyed; he lost his appetite, and became uniformly dull and sleepy. A discharge of matter also took place from his ear. The pain and stiffness at length extended precisely over half his face, accompanied by a throbbing in his ear, and a slight catching sometimes in his neck. He also complained of a dull heavy pain on the right side of his head, particularly affecting his ear and fore-head. In the course of the first month after his admission, he was cupped on the temples and between the shoulders two or three times; bled from the arm to ℥xx. and had, at three several times, six moxas applied on the affected side of the face; he took, also, some 5 gr. doses of calomel, followed by strong aperient medicine, by all which treatment he was so much weakened as to be overpowered by very slight exertion. However, after the cupping, he always felt relief and in some degree after the bleeding; and he thought the pain in his face a little better after the application of the moxas. On the whole, he was much freer from pain than when he came into the house.

"At this time, a seton applied in the nape of his neck, gave relief to his head, but the pain began to extend occasionally to the left side of his forehead. The throbbing and pain in his right ear continued much the same. Recovering from his state of extreme weakness, the pains in his face increased, and became again as bad as ever; a stiffness in his neck was also perceptible on the right side."

In the course of September, blisters behind the ear, antimonial ointment, and cuppings, were productive of temporary benefit; but, in October, there was costiveness, and the pain in his head was very severe. The latter was shaved, and moxas applied with some relief. Scruple doses of the carbonate of iron were now tried, but they aggravated the symptoms and were discontinued. On the 9th of November, it is reported (he had previously lost his voice) that " he *describes* his sensations as if an immense weight were placed upon his head, and when he moves it, or begins to doze, he experiences a dreadful whirling sensation. This he has felt, in some degree, ever since he came in, but it has increased greatly within these two days, so as to keep him constantly awake. His throat appears choked up with something which prevents his speaking, except in a whisper." The symptoms gradually increased, a great and augmenting discharge of mucus from the right nostril took place, and in February the patient died. The account of the sectio cadaveris is extremely brief, too much so, indeed, to convey much information respecting the actual character of the disease to the readers of the case. We must give it as we find it.

" The post-mortem examination discovered a large tumour arising from the dura mater, covering the middle division of the right side of the base of the skull, consisting of a number of encysted tubercles, filling up the hollow of the squamous portion of the temporal bone, thence (the bony matter, which would have otherwise stopped its progress, being destroyed) descending into the throat, and forming there a large conical pendulous projection. The front of the tumour blocked up the posterior entrance of the nostril, and its right side that of the corresponding eustachian tube."

The second case is one of a tumour, apparently, as far as we can judge from the history and symptoms, fungus hæmatodes of the testicle. As the patient, however, went out of the house before dissection could solve all doubts, we do not consider the case satisfactory enough to notice in detail.

III. CATARACT SUCCESSFULLY TREATED BY COUCHING.

W. Hill, æt. 38, entered the infirmary with hard lenticular cataracts, which prevented his distinguishing any object, but allowed him to discriminate easily between light and darkness. The application of the extract of belladonna produced great dilatation of the pupils, with increase of the power of vision. He had been subject to ophthalmia since his childhood, had lost all his eye-lashes, and his eye-lids were considerably thickened. The first symptoms of cataract took place about a year and a half previously. After a dose of calomel and an opening draught, the extract of belladonna was applied, and Mr. Fletcher immediately proceeded to the operation. He first punctured the eye with Saunders' needle, about three lines and a half behind the transparent cornea, and transfixing the lens obliquely in a horizontal direction, carried it away to the bottom of the eye by a circular motion. He then gradually withdrew the needle, rotating it gently, to free it from the lens. In the same way, he operated immediately afterwards on the right eye. Cold applications over the eyes, a dark room, and low diet, prevented any inflammation; the lenses remained depressed, and the patient was discharged, with restored sight, the third or fourth week after the operation; a very satisfactory result.

---

